# Patterns and Impact of Traumatic Brain Injury at King Abdulaziz Medical City in Jeddah, Saudi Arabia: A Retrospective Cohort Study

**DOI:** 10.7759/cureus.20246

**Published:** 2021-12-07

**Authors:** Fareeda S Alghamdi, Dania M Alsabbali, Yasmin H Qadi, Sarah M Albugami, Ahmed Lary

**Affiliations:** 1 College of Medicine, King Saud Bin Abdulaziz University for Health Sciences, Jeddah, SAU; 2 Neurological Surgery, National Guard Health Affairs, Jeddah, SAU

**Keywords:** subdural hematoma (sdh), fall from height, glasgow coma scale, motor vehicle accident, traumatic brain injuries

## Abstract

Objectives

The objectives of this study are to explore the most common causes, patterns, and severities of head traumas, to evaluate the outcomes of traumatic head injury (TBI) patients followed in the clinic, and to calculate the prevalence of admitted cases.

Methods

In our retrospective cohort study, we included all the cases of adults above 18 years old diagnosed with head traumas (171 patients). The inclusion criteria were patients who presented to the emergency department at National Guard Health Affairs (NGHA), Jeddah, Saudi Arabia from 2016 to 2020. Patients were categorized according to their Glasgow Coma Scale (GCS) score upon admission.

Results

Of the 171 patients in this study, 151 (88.3%) were males and 20 (11.7%) were females. The median age of our patients was 31 years. Most of the cases had no medical illnesses 124 (72.5%). The most common mechanism of injury was motor vehicle accidents (MVAs) in the majority of our cases (105, 61.4%), followed by falls from heights (34, 19.9%). The commonest computed tomography (CT) finding was subdural hematoma (47, 27.4%). The majority of the associated injuries were thoracic cases (43, 25.1%), followed by spinal (40, 23.4%). Most of the patients were admitted to NGHA (120, 70.2%), while the rest (51, 29.8%) were transferred from other hospitals. Of the total of 171 patients, 134 (78.4%) were treated conservatively. There were no associations between mortality nor length of stay and patients’ demographics, except for GCS on admission showed a significant p-value (<0.005).

Conclusion

In this study, it was found that the most common causes of TBI are MVAs followed by falls from heights. Therefore, preventive measures such as traffic safety rules need to be addressed.

## Introduction

Traumatic brain injury (TBI) is any interference with the normal function of the brain that can be caused by any force to the head the latter of which has been shown by Al-Habib et al. to result from mainly MVAs and pedestrian injuries [1,2]. It has been reported that the global incidence rate of TBI at 200 per 100,000 people annually, making it an essential topic of research [3]. Moreover, TBI is the leading cause of morbidity and mortality in Americans between 1 and 45 years old [4]. The most common cause of TBI is motor vehicle accidents (MVAs) contributing to 60% of all injuries followed by falls which account for 25%-30% of total injuries [5]. Similarly, a study conducted in Riyadh, Saudi Arabia revealed that the majority of injuries were caused by motor vehicle collisions and pedestrian injuries in 69.4% and 16.8%, respectively [2]. 

The types of TBI can be divided into two major categories which are primary and secondary injuries based on the timing of the insult. Primary injuries include those sustained at the time of the traumatic event. For example, vascular disruption, contusions, and diffuse axonal injury. Secondary injuries result from metabolic and physiologic changes that begin at the time of the initial injury and can last for hours and days. Secondary brain injuries include cerebral edema, alterations in cerebral blood flow, and intracranial hypertension [6]. Another way of categorizing TBI is by the level of severity using the Glasgow Coma Scale (GCS) score. The GCS score has been the most widely used clinical measure of the severity of injury in patients with TBI since 1974, and it is one of the parameters that will be assessed in this research [7].

Since King Abdulaziz Medical City is a level 1 trauma center, a large number of patients with TBI were available for this research study. These cases are either admitted cases or eligible transferred cases from other facilities. Therefore, we aim to explore the most common causes of TBIs at our institutions including MVAs, falls, direct trauma, and other possible causes. This will aid in implementing prevention programs for injury mechanisms in general including those with the highest prevalence and worst clinical outcomes. In addition, it will encourage building trauma centers in multiple areas with staff trained to deal with TBI.

## Materials and methods

Study area/settings

This retrospective review was a single-center study conducted in the Department of Neurosurgery, King Abdulaziz Medical City, Jeddah, Saudi Arabia. Data from 2016-2020 for all patients admitted to this department who fulfilled the inclusion and exclusion criteria were collected from the electronic database as IRB permission was taken, protocol number: NRJ21J/053/02. 

Study subjects

The included cases in this study were those who were admitted or transferred to neurosurgery due to head trauma at King Abdulaziz Medical City in Jeddah from 2016 to 2020. Patients who sustained injuries without head injury to the neurosurgery department, dead on presentation, or sustained injury during hospital stay due to other reasons were excluded. The study design was a retrospective cohort study.

Sample size

The requisite sample size was calculated using Rasoft software from the website www.rasoft.com/samplesize.html. The total number of TBI patients who presented to the ER from 2016 to 2020 is 500 patients. The required sample size was estimated at a 95% confidence interval with an estimated margin of error plus or minus 5%. The required minimum sample size was determined to be 2018. As our population is limited we included all patients that fit the criteria in the study.

Data collection methods, instruments used, measurements

Data included each patient's demographics, mechanism of injury, GCS score, clinical diagnosis, associated injuries, length of stay, function on discharge, and disposition.

Data management and analysis plan

All analyses were conducted using Statistical Package for the Social Sciences (SPSS) 2.0 (IBM Corp., Armonk, NY). A p-value <0.5 was considered significant. Qualitative variables were presented as frequency and percentages, while quantitative variables as +/- mean standard deviation. The Chi-square test was used to compare qualitative variables, while the T-test and analysis of variance (ANOVA) were used to compare quantitative variables. 

## Results

Among all cases who presented with TBIs between January 1, 2016 and December 31, 2020 to the National Guard Health Affairs (NGHA), a total of 171 cases met the inclusion criteria of the present study and were analyzed. The sample median age was 31 years old with an interquartile range (IQR) of 36 years. Of 171 patients, 151 males (88.3%). Most of the included cases (72.5%) had no medical illnesses. Among those who had comorbidities, 8.8% of cases were suffering from one chronic disease, while 18.7% had two or more chronic diseases which included diabetes mellitus, hypertension, cardiovascular diseases, osteoarthritis, thyroid diseases, malignancy, and psychiatric disorders. Motor vehicle accidents (61.4 %) dominated the mechanisms of injury in the current sample which was more than 60% of the mechanisms followed by injuries due to falls from height which was the case in 41.86% of the patients (Table 1). 

**Table 1:** Demographic characteristics of traumatic brain injury patients. IQR: interquartile range; MVA: motor vehicle accident.

n = 171	Median	IQR
Age (years)	31	36
	Frequency (n)	Percentage (%)
Gender		
Male	151	88.3
Female	20	11.7
Comorbidities		
None	124	72.5
One comorbidity	15	8.8
Two or more comorbidities	32	18.7
Mechanism of injury	Male	Female
MVA	91	14
Fall from heights	30	4
Hit by heavy object	5	0
Pedestrian	19	1
Other	6	1

TBIs were not the only injuries in these patients, since many individuals suffered from multiple other associated injuries. Thoracic injuries (25.1%) were the most common specified associated injuries. Additionally, spinal injuries were the second most prevalent associated injuries representing 23.4%. Moreover, other associated injuries were present in 72 cases (42.1%). Initial computed tomography (CT) findings were studied, and subdural Hematomas were prevalent in 53.2% of the 171 cases making it the most common CT finding in all patients of this study (Table 2).

**Table 2:** Associated injuries and CT findings in traumatic brain injury patients. EENT: eye, ear, nose and throat; CT: computed tomography. *This includes skull fractures, brain edema, and brain infractions.

n = 171	Frequency (n)	Percentage (%)
Associated injuries		
Thoracic injury	43	25.1
Spinal injury	40	23.4
Limb fracture	39	22.8
Abdominal injury	26	15.2
Facial fracture	19	11.1
Skull fracture	10	5.8
EENT injury	8	4.7
Other associated injuries	72	42.1
CT findings		
Isolated subdural hematoma	47	27.5
Isolated subarachnoid hemorrhage (SAH)	15	8.8
Isolated brain contusion	12	7
Isolated epidural hemorrhage	12	7
Isolated intracerebral hemorrhage (ICH)	4	2.3
Isolated brain hernia	1	0.6
Isolated intervertebral hemorrhage	1	0.6
Subdural hematoma + hernia + ICH	1	0.6
Subdural hematoma + SAH + ICH	1	0.6
Subdural hematoma + SAH + brain contusion	5	2.9
Subdural hematoma + ICH + brain contusion	2	1.2
Subdural hematoma + brain hernia	2	1.2
Subdural hematoma + brain hernia + ICH + SAH	1	0.6
Subdural hematoma + diffuse axonal injury	1	0.6
Subdural hematoma + epidural hematoma	9	5.3
Subdural hematoma + SAH	11	6.4
Subdural hematoma + SAH + interventricular hemorrhage	1	0.6
Subdural hematoma + brain contusion	2	1.2
Subdural hematoma + epidural hematoma + SAH + diffuse axonal injury	1	0.6
Subdural hematoma + epidural hematoma + SAH	1	0.2
Subdural hematoma + epidural hematoma + ICH	1	0.6
Subdural hematoma + SAH + intraventricular hemorrhage + ICH + brain hernia	1	0.6
Subdural hematoma + epidural hematoma + interventricular hemorrhage	1	0.6
Subdural hematoma + interventricular hemorrhage	1	0.6
Subdural hematoma + ICH	2	1.2
ICH + brain hernia	2	1.2
ICH + interventricular hemorrhage	1	0.6
ICH + SAH	2	1.2
Epidural hematoma + SAH	2	1.2
Epidural hematoma + SAH + ICH	2	1.2
Epidural hematoma + SAH + brain contusion	2	1.2
Epidural hematoma + brain contusion	1	0.6
Epidural hematoma + ICH	3	1.8
Epidural hematoma + brain hernia	1	0.6
SAH + brain contusion	6	3.5
SAH + ICH	1	0.6
SAH + ICH + interventricular hemorrhage	1	0.6
SAH + diffuse axonal injury	1	0.6
SAH + epidural hematoma + brain hernia + ICH	1	0.6
Other CT findings*	9	5.3

The majority of the cases (70.2%) were admitted to the NGHA through the emergency department while 29.8% were transferred from other sites of admission. According to the records, the median length of stay of these 171 patients was 10 days (IQR = 27 days). Among all 171 patients, most patients (63.7%) were diagnosed with intracranial hematoma based on initial CT findings or magnetic resonance imaging (MRI) done later on admission. Regarding the treatment, the larger proportion of these patients (78.4%) were treated conservatively, while 15.20% underwent neurosurgical intervention for their operative TBI. Furthermore, 84.2% of the patients were alive after receiving treatment; however, the other 15.2% of patients, unfortunately, expired. Table 3 shows detailed data of the patients’ course of admission. GCS score at the time of diagnosis and discharge were summarized in Table 4.

**Table 3:** Course of patients after admission. IQR: interquartile range; NGHA: National Guard Health Affairs Hospital. *Home without healthcare. **This includes: brain contusion, brain herniation, and diffuse axonal injury.

n = 171	Median	IQR
Length of stay (days)	10	27
	Frequency (n)	Percentage (%)
Admission source (n=171)		
NGHA	120	70.2
Transferred	51	29.8
Admission site (n=141)		
Neurosurgery	57	40.4
Care under other specialties	84	59.6
Disposition (n=132)		
Nursing home	2	1.5
Home with health care	16	12.1
Home*	114	86.4
Diagnosis		
Traumatic brain injury	13	7.6
Intracranial hematomas/hemorrhage	109	63.7
Other diagnoses**	49	28.7
Treatment		
Conservative	134	78.4
Operative	37	21.6
Outcome		
Alive	144	84.2
Deceased	27	15.8

**Table 4:** GCS score of traumatic brain injury patients. GCS: Glasgow Coma Scale.

	GCS score on admission		GCS score on discharge	
N = 171	Frequency	Percentage	Frequency	Percentage
15 – 13	60	35.1	95	55.6
12 – 9	26	15.2	16	9.4
< 9	72	42.1	15	8.8
Unknown	13	7.6	45	26.2

Demographic characteristics and other factors such as the mechanism of injury and GCS score were studied to search for an association with mortality. Although differences were observed between many groups, these differences were not statistically significant. Table 5 illustrates the distribution of some of these factors. Moreover, the length of stay was investigated. Difference in length of stay based on numerous factors such as age, gender, GCS score and mechanism of injury were studied. The GCS score on admission was associated with a difference in the length of stay and this difference achieved a statistical significance. However, associations between the length of stay and other factors were not statistically significant (Table 6).

**Table 5:** Association between mortality and patients demographics. *Mann Whitney U test. **Kruskal-Wallis H.

n = 171	Alive	Deceased	p-value
Age (years)			0.967*
≤ 20	27	5 (3%)	
21 – 40	62	11 (6%)	
≥ 40	55	11 (6%)	
Gender			0.353**
Male	126	25 (15%)	
Female	18	2 (1%)	
Comorbidities			0.535*
None	46	7 (4%)	
Present	98	20 (12%)	

**Table 6:** Difference in length of stay between groups of traumatic brain injury patients. MVA: motor vehicle accident; GCS: Glasgow Coma Scale. *Mann Whitney U test. **Kruskal-Wallis H.

Variable	Groups	Mean rank	U/H	p-value
Age (years)	≤ 20	77.11	1.258	0.533**
	21 – 40	88.78		
	≥ 40	85.99		
Gender	Male	83.20	1155.500	0.095*
	Female	102.73		
Comorbidities	None	87.21	2900.000	0.5*
	Present	81.72		
Mechanism of injury	MVA	85.40	2.504	0.644**
	Fall from height	81.62		
	Hit by heavy object	62.60		
	Pedestrian	97.50		
	Other	87.93		
GCS score on admission	15 – 13	60.98	24.009	<0.005**
	12 – 9	92.19		
	< 9	99.61		
	Unknown	108.19		

## Discussion

This study was a retrospective analysis of cases presenting with TBI. TBI incidences differ greatly between and within countries due to a lack of data on an exact definition of head injury, subtypes, etiology, methodological concerns, and outcomes. The study's findings revealed that the most common cause of TBI was a motor vehicle accident (MVA) followed by fall from heights. Similar findings were observed in studies done in other Middle Eastern nations such as the United Arab Emirates and Qatar, indicating that MVAs are the leading cause of trauma in TBI [8]. Research performed in the United States in 2007 and 2013 found that falls from great heights and assault were the top causes of TBI, while MVAs remained the leading cause of mortality following TBI [9,10]. In the present study, the majority of the patients presenting with TBI were males, and the leading cause among males was MVAs, thus emphasizing the importance of preventive measures and appropriated public health programs, including traffic safety rules. These findings are in accordance with previous reports from other provinces in Saudi Arabia [11,12]. In our study, more than one-fourth of patients were suffering from chronic diseases, and it has been reported by others that the presence of diabetes mellitus, hypertension, and cardiovascular problems could promote poor clinical outcomes in TBI patients [13-15]. 

The pathophysiology of TBI is a complex process that begins with primary and secondary injuries resulting in short or long-term brain impairments. The primary impairment is directly related to the brain's major external impacts causing compression and shearing of adjacent tissues with or without loss of consciousness. The secondary injury occurs minutes to days after the main impact and is caused by a molecular, chemical, and inflammatory cascade resulting in cranial and systemic complications [16]. Brain injuries incurred in MVAs, assaults, or falls from heights typically result in damage to multiple body sites, including the spine and vertebrae, and these are found to be the prime cause of mortality and disability [17]. The findings of our study showed thoracic injury and spine injury were the more commonly associated injuries seen in these patients. One report showed that in an overall major trauma outcome study in cases with severe extracranial injury (SEI) alone, the mortality rate was 8.3%; it was three times higher in the SEI with head injury group (14.5%) than in the non-head injury patients (5.1%) [18]. In another report, moderate brain injury on its own had a 4.2% mortality rate. When combined with extracranial injury the risk of death was double that due to extracranial injury alone [19]. Patients with injury in extracranial sites could have a massive extracranial site hemorrhage, which could result in a coagulopathy or decreased cerebral blood flow both of which could result in secondary brain damage [20,21]. We observed a mortality rate of 15.8%, even though there was no significant association seen with age, gender, and presence of comorbidities. Studies show that older age groups have significantly higher mortality rates after TBI incidence [22,23]. A study done by Munivenkatappa et al. reported that the mortality rate among female patients was significantly higher compared to males (3.4% vs. 1.6%) [23]. Thus it is important to consider these factors to predict the outcomes that can help doctors to take precautionary measures while managing patients with TBI. Patients suspected of having suffered a TBI should be transferred directly to a Level I or II Trauma Care Center (TCC) unless they require life-saving stabilization in a non-TCC [24]. 

Coma severity is measured using the GCS score, which may be used to a wide range of acute medical and trauma patients, and it should be determined soon after arrival or within less than one hour after arrival to the hospital. A GCS score of 15-13 is considered as a mild, 9-13, a moderate and 3-8, a severe head injury [25]. In our study, patients with severe and moderate GCS scores on admission had significantly longer hospital stays than those with mild GCS scores. However, no statistically significant differences in the length of hospital stay were associated with age, gender, comorbidities, and mechanism of injury. Patients with severe GCS scores were found to have more poor clinical outcomes and higher mortality rates [26]. Also, it is reported that a 100% mortality was seen in cases with fixed and dilated pupils with severe GCS scores [27,28]. A GCS score of ≥ 13 has been reported to have better clinical outcomes following TBI, and individuals with GCS scores ≤13 must be examined frequently and constantly monitored since these patients may require emergency intubation [29].

Our study was cross-sectional hospital-based with a retrospective design; hence could have some limitations of its own. Our study's data collection, refining, and quality control techniques are comparable to those of prior databases; retrospective investigations still carry their own inherent biases and these cannot be ignored completely. Second, as this is a hospital-based study, there may be a potential for referral bias. Also, we didn't record the type of interventions these patients received while they were in the hospital, which might affect their recovery. Thirdly, "milder" head injuries that didn't require hospitalization might be missed since the database includes people who were admitted or died in the emergency department.

## Conclusions

In this study, it was found that the most common causes of TBI are MVAs followed by falls from height. Therefore, preventive measures such as traffic safety rules need to be addressed. Furthermore, TBI is not broadly studied in our region, thus, we recommend conducting further research in this area to highlight the importance of implementing safety measures and building trauma centers that will aid in better patients' outcome in terms of morbidity and mortality.
